# The investigation of implicit Theory of Mind in patients with schizophrenia – a whole brain fMRI study

**DOI:** 10.1192/j.eurpsy.2022.522

**Published:** 2022-09-01

**Authors:** E. Varga, T. Bugya, A. Hajnal, T. Tényi, R. Herold

**Affiliations:** 1 University of Pécs, Medical School, Department Of Pediatrics, Pécs, Hungary; 2 University of Pécs, Department Of Cartography And Geoinformatics, Pécs, Hungary; 3 University of Pécs, Department Of Psychiatry And Psychotherapy, Pécs, Hungary

**Keywords:** schizophrénia, Neuroimaging, Theory of Mind, fMRI

## Abstract

**Introduction:**

Theory of Mind is the ability to attribute mental states to others. Investigations have distinguished implicit and explicit forms of ToM. It is known, that patients with schizoprenia have deficits in their explicit ToM, and they also show altered brain activations during examining explicit ToM.

**Objectives:**

In this study our aim was to investigate the underlying neural substrates of implicit ToM in patients with schizophrenia with fMRI.

**Methods:**

Seven healthy subjects and two patients with first episode schizophrenia were involved. We used: false belief condition and control condition. All movies consisted of a belief formation phase and an outcome phase. The belief formation phase started with an agent placing a ball on a table in front of an occluder. Then the ball rolled behind the occluder. The movies could continue in different ways leading to a true or false belief. At the end of each movie, the agent reentered the scene and the occluder was lowered. In the outcome phase the ball was either present or absent behind the occluder. The control conditions started with a ball rolled behind the occluder on a table ended up with two different ways as the ball was either present or absent behind the occluder. There was no agent in the control movies.

**Results:**

We found that healthy subjects activated significantly stronger the left lingual gyrus as well as the right temporoparietal junction.

**Conclusions:**

Our findings suggest deficits in implicit ToM in schizophrenia and our findings also might help to clarify the underlying neural substrates of implicit ToM.

**Disclosure:**

This research project was supported by the KTIA-13-NAP-A-II/12 (2018–2022) and the Hungarian National Excellence Centrum Grant 2018–2019.

